# Mitochondrial complex I deactivation is related to superoxide production in acute hypoxia

**DOI:** 10.1016/j.redox.2017.04.025

**Published:** 2017-04-21

**Authors:** Pablo Hernansanz-Agustín, Elena Ramos, Elisa Navarro, Esther Parada, Nuria Sánchez-López, Laura Peláez-Aguado, J. Daniel Cabrera-García, Daniel Tello, Izaskun Buendia, Anabel Marina, Javier Egea, Manuela G. López, Anna Bogdanova, Antonio Martínez-Ruiz

**Affiliations:** a Servicio de Inmunología, Hospital Universitario de La Princesa, Instituto de Investigación Sanitaria Princesa (IIS-IP), E-28006 Madrid, Spain; b Departamento de Bioquímica, Facultad de Medicina, Universidad Autónoma de Madrid (UAM) and Instituto de Investigaciones Biomédicas Alberto Sols, E-28029 Madrid, Spain; c Instituto Teófilo Hernando, Departamento de Farmacología y Terapéutica, Facultad de Medicina, Universidad Autónoma de Madrid (UAM), Instituto de Investigación Sanitaria Princesa (IIS-IP), E-28029 Madrid, Spain; d Servicio de Proteómica, Centro de Biología Molecular “Severo Ochoa (CBSMO), Consejo Superior de Investigaciones Científicas (CSIC) – UAM, E-28049 Madrid, Spain; e Unidad de Investigación, Hospital Santa Cristina, Universidad Autónoma de Madrid (UAM), Instituto de Investigación Sanitaria Princesa (IP), E-28009 Madrid, Spain; f Institute of Veterinary Physiology, Vetsuisse Faculty and Zurich Center for Integrative Human Physiology (ZIHP), University of Zurich, CH-8057 Zurich, Switzerland; g Centro de Investigación Biomédica en Red de Enfermedades Cardiovasculares (CIBERCV), Spain

**Keywords:** Hypoxia, Oxygen sensing, Superoxide, Mitochondrial complex I, Redox signalling

## Abstract

Mitochondria use oxygen as the final acceptor of the respiratory chain, but its incomplete reduction can also produce reactive oxygen species (ROS), especially superoxide. Acute hypoxia produces a superoxide burst in different cell types, but the triggering mechanism is still unknown. Herein, we show that complex I is involved in this superoxide burst under acute hypoxia in endothelial cells. We have also studied the possible mechanisms by which complex I could be involved in this burst, discarding reverse electron transport in complex I and the implication of PTEN-induced putative kinase 1 (PINK1). We show that complex I transition from the active to ‘deactive’ form is enhanced by acute hypoxia in endothelial cells and brain tissue, and we suggest that it can trigger ROS production through its Na^+^/H^+^ antiporter activity. These results highlight the role of complex I as a key actor in redox signalling in acute hypoxia.

## Highlights

• Complex I is involved in the superoxide burst produced by cells in acute hypoxia.
• Complex I is deactivated in acute hypoxia.
• Deactive complex I is involved in superoxide production in acute hypoxia, probably through its Na^+^/H^+^ antiporter activity.
• Complex I deactivation occurs in brain tissue hypoxia *ex vivo* and *in vivo*.

## Graphical abstract

**Figure:**
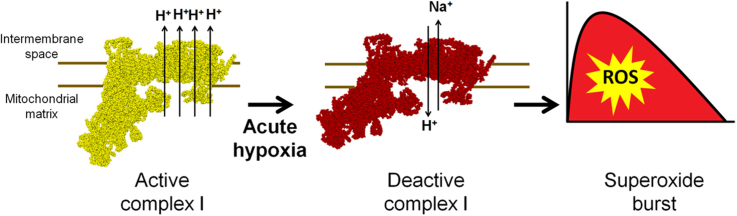

## 1 Introduction

Eukaryotic organisms use oxygen (O_2_) as the final electron acceptor in the mitochondrial electron transport chain, producing water (H_2_O) and driving the production of the high-energy molecule ATP through oxidative phosphorylation (OXPHOS). The OXPHOS system is located in the mitochondrial inner membrane and is composed of five complexes which couple the pumping of H^+^ to the transfer of electrons from different substrates, such as NADH (oxidised by complex I) and succinate (oxidised by complex II). The difference in charges and pH generated across the mitochondrial inner membrane establish the mitochondrial membrane potential (ΔΨmt) and the pH gradient (ΔpH), respectively. Both parameters determine the protonmotive force (Δµmt) essential to drive OXPHOS. A series of reactive oxygen species (ROS) is also formed from the incomplete reduction of O_2_ during respiration [1], [2]. ROS can oxidise the majority of cellular components including nucleic acids, lipids and proteins, and are known to be associated with cell damage, particularly in conditions of oxidative stress [3]. Mitochondrial ROS are involved in many pathological scenarios [4] such as stroke [5], cancer [6], Parkinson's [7], Alzheimer's [8] or cardiovascular diseases, where its overproduction may contribute to disease progression. However, it is acknowledged that mitochondrial ROS also act as second messengers in cell signalling processes in a variety of physiological conditions [9], [10], [11], [12], [13], [14], [15].

Among the five complexes comprising OXPHOS, complex I is the largest and performs a reversible NADH-ubiquinone oxidoreductase reaction coupled to pumping four H^+^ across the mitochondrial inner membrane. Complex I is formed by a hydrophilic arm which incorporates one flavin mononucleotide (FMN) and eight iron-sulfur clusters involved in electron transfer across this structure. The hydrophilic domain is attached to a hydrophobic arm involved in the H^+^-pumping function of the complex. Energy transfer to the hydrophobic domain occurs through the stabilization of the oxidised quinone in the ubiquinone-binding site which allows a series of conformational rearrangements necessary for H^+^ pumping [16]. Complex I can also undergo a deactivation process named active/‘deactive’ transition (A/D transition) which implies a switch from a NADH-ubiquinone oxidoreductase activity to a Na^+^/H^+^ antiporter through its hydrophobic arm [17], [18]. Importantly, deactivation includes a series of conformational changes in which the Cys39 of the complex I subunit ND3 becomes exposed. This exposure has been used as a marker of deactivation [19], [20]. In addition, complex I can be modulated by proteins and lipids [21] whose deregulation can lead to pathophysiological scenarios. Among them, a genetic variant of Parkinson's disease involves the mutation of the PTEN-induced putative kinase (PINK1) gene which has been associated with lower complex I activity and increased ROS production [22], [23].

Mitochondrial complex I is also a major site of superoxide anion production in the mitochondria [1], [24] through both forward and reverse reactions (electron transfer from NADH to ubiquinone, or from reduced ubiquinone to NAD^+^, respectively). The reverse reaction or reverse electron transfer (RET) needs a large pool of reduced ubiquinone which is normally generated from succinate oxidation through mitochondrial complex II, can be inhibited by rotenone and is dependent on high ΔΨmt [25], [26]. RET has been implicated in exacerbated ROS production in reperfusion after ischemia [27], [28].

Cells are frequently subjected to changes in oxygen availability and must adapt in order to survive. A decrease in oxygenation (hypoxia) induces a series of acute and long-term cellular, tissue-specific and systemic adaptive responses [29]. Both types of responses have been linked to the production of ROS. Whether ROS generation increased or decreased in hypoxia was strongly debated for years [30], [31]. We have recently described that superoxide anion is produced in the first minutes of hypoxia by the mitochondria in different cell types, and correlates in endothelial cells with the oxidation of protein thiols [32], [33].

More recently, it has been described that complex I is involved in the specialized acute response to hypoxia that takes place in the carotid body [34], where it triggers a ROS signal that activates ion channels provoking the release of neurotransmitters and hyperventilation [35]. Herein, we describe that complex I is involved in the ROS burst produced in acute hypoxia, in endothelial cells but also in brain tissue, and the mechanism by which complex I may be involved in triggering this response.

## 2 Materials and methods

### 2.1 Animals, cell culture and transfection

All animal experiments were performed following the Guide for the Care and Use of Laboratory Animals and were previously approved by the institutional ethics committee of the Universidad Autónoma de Madrid, Spain, according to the European guidelines for the use and care of animals for research in accordance with the European Union Directive of 22 September 2010 (2010/63/UE) and with the Spanish Royal Decree of 1 February 2013 (53/2013). All efforts were made to minimize the number of animals used and their suffering.

Cells were routinely maintained in cell culture incubators (95% air, 5% CO_2_ in gas phase, 37 °C). Bovine aortic endothelial cells (BAECs) were isolated as previously described [36] and cultured in RPMI 1640 supplemented with 15% heat-inactivated foetal bovine serum (FBS), 100 U/mL penicillin and 100 μg/mL streptomycin. BAECs were used between passages 3–9. Endothelial morphology was assessed by visual inspection.

Transfection of 30 nM siRNA or 0.25 µg pHyPer-Cyto (CytoHyPer), C199S pHyPer-Mito (mitosypHer) or C199S pHyPer-Cyto (cytosypHer) vector DNA per 0.8 cm^2^ well was carried out using Lipofectamine 2000 (Invitrogen) according to the manufacturer's instructions. Experiments were carried out 48 h after transfection.

### 2.2 siRNA preparation

Doubled-stranded siRNAs against bovine NDUFS4, NDUFS2 and PINK1 were designed and purchased from Integrated DNA Technologies (NDUFS4 sense sequence GCUGCCGUUUCCGUUUCCAAGGUUUTT; NDUFS2 sense sequence TCGGACAGTCGACATTGGGATT; PINK1 sense sequence GGCUGCUAAUGUGCUUCAUUU). siSCR was purchased from Santa Cruz Biotechnology.

### 2.3 Detection of superoxide by fluorescence microscopy in fixed cells

BAECs were seeded on glass coverslips one day before experimentation. In some experiments, 1 µM rotenone or 1 µM carbonyl cyanide-p-trifluoromethoxyphenylhydrazone (FCCP) was added 30 min before experimentation and maintained during the experiment. For treatments in hypoxia, all the solutions were pre-equilibrated in hypoxic conditions before use; plated cells were introduced in an Invivo2 400 workstation (Ruskinn) set at 1% O_2_, 5% CO_2_, 37 °C, and incubated for the indicated times (0, 15, 30, 45 and 60 min) in fresh medium, washed three times with Hank's Balanced Salt Solution with Ca^2+^/Mg^2+^(HBSS+Ca/Mg) and incubated with 5 µM dihydroethidium (DHE) in HBSS+Ca/Mg for 10 min in the dark. Excess probe was removed by three washes with HBSS+Ca/Mg, the cells were fixed with 4% paraformaldehyde (PFA), and incubated in the dark at 4 °C for 15 min. After fixation, the cells were again washed three times with HBSS+Ca/Mg and coverslips were placed on slides. For normoxic treatments, the medium was changed for fresh normoxic medium, and treated as hypoxic cells, but in a standard cell incubator. Images (three images per each coverslip; the number of independent experiments is described in the figure legends) were taken with a Leica DMR fluorescence microscope with a 63x objective, using the 546-12/560 excitation/emission filter pairs and quantified using ImageJ software (NIH). The same threshold was set for all the images and the mean value from histograms was averaged for the three images of each coverslip.

### 2.4 Detection of intracellular ROS by live imaging fluorescence microscopy

BAECs were seeded in 6-well plates one day before experimentation. Plated cells were washed three times with HBSS+Ca/Mg and incubated with DHE for 20 min at 37 °C in the dark. 1 µM FCCP was also added at this time and maintained during the experiment. DHE was then washed out and new HBSS+Ca/Mg was added. After this time, the plate was placed into a Leica DM 16000B fluorescence microscope equipped with a Leica DFC360FXcamera, an automated stage for live imaging and a thermostated hypoxic cabinet. The planes were focused for image capture, and images were taken with a 20x objective every 2 min during 40 min, providing a total of 21 cycles. Normoxia experiments started and ended at 20% O_2_ and 5% CO_2_, whereas hypoxia experiments started at 20% O_2_ and 5% CO_2_ and then were switched to 2% O_2_ and 5% CO_2_ in cycle 2 (due to technical limitations of the hypoxia cabinet, it was not possible to set O_2_ concentration below 2%). The excitation/emission filter pair used was 546-12/560. Images were quantified with Image J software. Three independent experiments were performed for each condition. For each experiment and condition, four regions of interest (ROIs) were created, each ROI surrounding an individual cell, and the mean fluorescence of each ROI for each time cycle was collected. In some analyses, for each experiment and condition, four identical linear ROIs were created and the maximum peak value of cycles 0, 5, 10, 15 and 20 were collected for each ROI.

### 2.5 Detection of intracellular ROS and intramitochondrial pH by live imaging confocal microscopy

To detect intramitochondrial pH, BAECs were transfected with the ratiometric probe mitosypHer in 8-well plates two days before the experiment. 1 µM rotenone or 1 µM FCCP were added 30 min before and maintained during the rest of the experiment. The plate was then placed into a Leica SP-5 confocal microscope, an automated stage for live imaging and a thermostated hypoxic cabinet. The planes were focused for image capture and images were taken with a 63x objective every 5 min during 30 min. Normoxia experiments started and ended at 20% O_2_ and 5% CO_2_, whereas hypoxia experiments started at 20% O_2_ and 5% CO_2_ and then were switched to 1% O_2_ and 5% CO_2_ in cycle 1. Excitation was performed with a 405 diode laser for 405 nm line and Ar/Kr for 488 nm line and fluorescent emission was recorded at 515–535 nm range. To assess mitochondrial colocalization, transfected cells were incubated with 25 nM MitoTracker CMTMRos for 20 min at 37 °C in HBSS+Ca/Mg in the dark, washed again three times with HBSS+Ca/Mg and fixed with 4% PFA; samples were excited with a Ar/Kr laser using the 488 nm line for mitosypHer and a He/Ne laser using the 543 nm line for MitoTracker. Fluorescence emission of mitosypHer was detected in 515–535 nm range and MitoTracker in 575–590 nm range.

For intracellular ROS detection, BAECs were transfected with the cytosolic version of HyPer (CytoHyPer) following the same procedure for live imaging as with mitosypHer.

Images were quantified with ImageJ software. Three or four independent experiments were performed for each condition. For each experiment and condition in loaded cells, four identical linear regions of interest (ROIs) were quantified, and for each time point the mean of these ROIs was obtained.

### 2.6 Submitochondrial particles (SMPs) isolation and blue native polyacrylamide gel electrophoresis (BN-PAGE)

BAECs were washed twice in ice-cold PBS, scraped off the plate and centrifuged for 5 min 600*g* at 4 °C. To obtain SMPs, cells were resuspended in 200 µL of PBS, mixed with 200 µL of 8 mg/mL digitonin and incubated for 10 min on ice. After this, 1 mL PBS was added and the samples were centrifuged 5 min at 10,000*g*, 4 °C. The resulting pellet of SMPs was washed and resuspended in 100 µL of 1.5 M aminocaproic acid, 50 mM Bis-Tris/HCl pH 7.0. Protein concentration was quantified by BCA assay. SMPs were centrifuged 2 min 13,500*g* at 4 °C and the pellet was resuspended at 10 µg/µL with 1.5 M aminocaproic acid, 50 mM Bis-Tris/HCl pH 7.0. SMPs were solubilized with 4*g* of digitonin per gram of protein, incubated 5 min on ice and centrifuged 30 min 16,000*g* at 4 °C. Supernatant was collected and mixed with sample buffer (Coomassie brilliant blue G-250 5% in 1 M aminocaproic acid solution). For each sample, 100–150 µg was loaded and run on a 3–20% gradient BN-PAGE gel as described [37]. Gel transfer was performed onto PVDF membranes, which were then washed with methanol for 3 min before western blotting.

### 2.7 Western blot analysis

Protein samples were extracted with non-reducing Laemmli buffer without bromophenol blue and quantified by the BCA assay. Extracts were then loaded onto 10% standard polyacrylamide gel electrophoresis after adding 5% 2-mercaptoethanol, and transferred to nitrocellulose membranes or PVDF membranes for BN-PAGE. The following antibodies were used: monoclonal anti-HIF-1α antibody (#MAB1536; R&D Systems), monoclonal anti-NDUFS4 antibody (ab87399; Abcam), monoclonal anti-NDUFS2 antibody (ab110249; Abcam), anti-NDUFB6 antibody (16037-1-ap, Proteintech), anti-ubiquinol-cytochrome *c* reductase core protein I antibody (ab110252; Abcam), anti-PINK1 (sc-33796, Santa Cruz Biotechnology) and monoclonal anti-α-tubulin antibody (T6199, Sigma). Antibody binding was detected by chemiluminescence with species-specific secondary antibodies labelled with horseradish peroxidase (HRP), and visualized on a digital luminescent image analyzer (Fujifilm LAS-4000).

### 2.8 Fluorescent labelling of ND3 Cys-39 from isolated mitochondrial membranes

For cell extracts and *ex vivo* samples, the procedure previously described for SMPs preparation was used. For *in vivo* samples, brain mitochondria isolation was performed using the Mitochondrial Isolation Kit for tissue (ab110168; Abcam) according to the manufacturer's protocol. Briefly, brain tissue was washed and minced in Isolation Buffer and cells were disrupted using a Dounce tissue grinder pestle (Sigma). Then, homogenized tissue was centrifuged at 1000*g* for 10 min at 4 °C and the supernatant was centrifuged at 12,000*g* for 15 min at 4 °C, yielding the enriched mitochondria and cytosol fractions in the pellet and the supernatant, respectively. SMP or mitochondrial protein amount was determined by the BCA assay and then proteins were solubilized with 4*g* of digitonin per gram of protein, incubated 5 min on ice and centrifuged 30 min at 16,000*g*, 4 °C. Samples from cell cultures were split into two parts, one part was incubated at 37 °C for 60 min to fully deactivate complex I and the other part was kept on ice. Samples were then incubated with Bodipy-TMR C5-maleimide (Invitrogen) for 20 min at 15 °C in the dark; then, 1 mM cysteine was added and the samples were further incubated for 5 min. After this time, the samples were precipitated twice with acetone, centrifuged at 9500*g* for 10 min at 4 °C in the dark, and the resulting pellet was resuspended in non-reducing Laemmli loading buffer. For each sample, 100 μg was loaded onto 10% Tricine-SDS-PAGE gels as previously described [38]. Total protein staining was performed with Sypro Ruby (Invitrogen) following the manufacturers' instructions. The images of the different fluorophores were obtained using a digital fluorescent image analyzer (Fujifilm LAS-4000). Images were quantified using ImageQuant TL7.0 software.

### 2.9 Protein mass spectrometry analysis

After drying, electrophoretic bands were cut in pieces, destained in acetonitrile: water (ACN:H_2_O, 1:1), reduced with 10 mM DTT for 1 h at 56 °C, alkylated with 50 mM iodoacetamide for 1 h at room temperature in the dark and digested *in situ* with sequencing grade trypsin (Promega, Madison, WI) as described by Shevchenko et al. [39] with minor modifications. The gel pieces were shrunk by removing all liquid using sufficient ACN. Acetonitrile was pipetted out and the gel pieces were dried in a speedvac. The dried gel pieces were re-swollen in 50 mM ammonium bicarbonate (AB) pH 8.8 with 12.5 ng/µL trypsin for 1 h in an ice-bath. The digestion buffer was removed and gels were covered again with 50 mM AB and incubated at 37 °C for 12 h. Digestion was stopped by the addition of 1% TFA. Whole supernatants were dried down and then desalted onto ZipTip C18 Pipette tips (Millipore) until the mass spectrometric analysis.

The desalted protein digest was dried, resuspended in 10 µL of 0.1% formic acid and analyzed by RP-LC-MS/MS in an Easy-nLC II system coupled to an ion trap LTQ-Orbitrap-Velos-Pro mass spectrometer (Thermo Scientific), as previously described [40] with minor modifications. The peptides were concentrated (on-line) by reverse phase chromatography using a 0.1 mm×20 mm C18 RP precolumn (Proxeon), and then separated using a 0.075 mm×250 mm C18 RP column (Proxeon) operating at 0.3 µL/min. Peptides were eluted using a 240-min dual gradient from 5% to 25% solvent B in 180 min followed by gradient from 25% to 40% solvent B over 240 min (Solvent A: 0.1% formic acid in water, solvent B: 0.1% formic acid, 80% acetonitrile in water). ESI ionization was carried out using a Nano-bore emitters Stainless Steel ID 30 µm (Proxeon) interface.

The mass spectrometer was operated in the selected MS/MS ion monitoring mode (SMIM mode [41]). In this mode, the LTQ-Orbitrap-Velos-Pro detector was programmed to perform, along the same entire gradient, a continuous sequential operation in the MS/MS mode on the doubly or triply charged ions corresponding to the peptide selected previously from the theoretical prediction.

Peptides were detected in survey scans with a mass range of 400–1600 u (in mass-to-charge ratio units, *m/z*), followed by ten data-dependent MS/MS scans (Top 10), using an isolation width of 2 u (*m/z*), normalized collision energy of 35%, and dynamic exclusion applied during 30 s periods. The Orbitrap resolution was set at 30,000. Peptide identification from raw data was carried out using the SEQUEST algorithm (Proteome Discoverer 1.4, Thermo Scientific). Database search was performed against handmade database and search against decoy database (integrated decoy approach) using false discovery rate (FDR) <0.01. The following constraints were used for the searches: tryptic cleavage after Arg and Lys, up to two missed cleavage sites, and tolerances of 20 ppm for precursor ions and 0.8 Da for MS/MS fragment ions, and the searches were performed allowing optional Met oxidation, Cys carbamidomethylation and Cys N-ethylmaleimide modification.

### 2.10 Preparation of mouse hippocampal slices

Three-month-old C57BL/6 mice were anesthetized with 1.5% isoflurane in oxygen under spontaneous respiration, then decapitated and forebrains were rapidly removed from the skull and placed into ice-cold Krebs bicarbonate dissection buffer (pH 7.4), containing (in mM): NaCl 120, KCl 2, CaCl_2_ 0.5, NaHCO_3_ 26, MgSO_4_ 10, KH_2_PO_4_ 1.18, glucose 11 and sucrose 200. The hippocampi were dissected, and slices (250-µm thick) were prepared using a McIlwain Tissue Chopper. Then, the slices were transferred to vials containing sucrose-free dissection buffer to allow tissue recovery from slicing trauma before experimentation (equilibration period). Both solutions were gassed with 5% CO_2_ at least 30 min before use to ensure pH 7.4.

Hippocampal slices were placed into an Invivo2 400 workstation (Ruskinn) set at 1% O_2_, 5% CO_2_, 37 °C, incubated for 30 min, disaggregated in PBS and SMPs extracted as previously described. For western blot analysis, slices were incubated for 4 h and lysed in non-reduced bromophenol-free Laemmli buffer inside the chamber.

### 2.11 Detection of superoxide in hippocampal slices by confocal microscopy

For treatments in hypoxia, all solutions were pre-equilibrated to hypoxic conditions before use; when necessary, 10 µM antimycin A was added 30 min before the experiment and maintained during the rest of the procedure. Hippocampal slices were placed into an Invivo2 400 workstation (Ruskinn) set at 1% O_2_, 5% CO_2_, 37 °C, and incubated for 30 min in fresh medium, washed three times with HBSS+Ca/Mg and incubated with 5 µM DHE for 10 min in the dark. After incubation, excess probe was removed by three washes in HBSS+Ca/Mg, slices were fixed with 4% paraformaldehyde, and incubated in the dark at 4 °C for 15 min. After fixation, wells were washed again three times with HBSS+Ca/Mg and the slices placed on slides with coverslips on top. For normoxic treatments, medium was exchanged for fresh normoxic medium, and treated as above, but in a standard cell incubator. Images of CA1 region of hippocampal slices were taken with a Leica SP-5 confocal microscope with a 40x objective. Samples were excited with aAr/Kr laser using the 488 nm line for hydroxyethidium (OH-Eth) and 496 nm line for ethidium (Eth). Fluorescence emission of OH-Eth was detected at 560–570 nm and Eth at 570–600 nm following previously-reported methods [42]. Three-dimensional image stacks were processed using ImageJ software. For each stack, the background was subtracted from the fluorescence intensity of the CA1 region, and two hippocampal slices were averaged in each independent experiment.

### 2.12 *In vivo* photothrombotic stroke

Three-month-old male C57BL/6 mice (30–35 g) were anesthetized with 1.5% isoflurane in oxygen under spontaneous respiration. Mice were then placed in a stereotaxic frame (David Kopf Instruments, Tujunga, CA, USA) and physiological temperature (37±0.5 °C) was maintained by a servo-controlled rectal probe heating pad (Cibertec, Madrid, Spain). A small incision in the midline was made and, after removal of the periosteum, bregma and lambda points were identified. A cold light (Zeiss KL 1500 LCD, Jena, Germany) was centred using a micromanipulator at 0.2 mm posterior and 1.5 mm lateral to bregma on the right side using a fiber optic bundle of 2 mm in diameter. One milligram of the photosensitive dye Rose Bengal (Sigma Aldrich, St. Louis, MO, USA) dissolved in sterile saline (0.1 mL) was injected i.p. and 5 min later the brains were illuminated during 20 min. After completion of the surgical procedures, the incision was sutured and the mice were allowed to recover for 24 h.

Twenty-four-hours after stroke induction, mice were anesthetized and sacrificed by decapitation and brains were quickly removed. Then, coronal sections of 1-mm-thickness were cut and slices were incubated in a 2% solution of triphenyltetrazolium chloride and then fixed in a buffered formalin solution. Infarcted tissue was defined by the unstained area.

### 2.13 Statistics

Normality and homoscedasticity tests were carried out before applying parametric tests. For comparison of multiple groups, we performed one-way ANOVA followed by Tukey test for all the groups of the experiment. For comparison of two groups, we used Student's two-tailed *t*-test; when the data did not pass the normality test, we used a non-parametric *t*-test (Mann-Whitney *U* test). Variations were considered significant when the p value was less than 0.05. Statistical analysis was performed with SigmaPlot 11.0 software.

Estimated Pearson and Mander's correlation coefficients for colocalisation of fluorescent signals in Fig. 6b were calculated using colocalisation plug-in of Image J software.

## 3 Results

### 3.1 ROS increase in acute hypoxia is dependent on complex I function

**Fig. 1:**
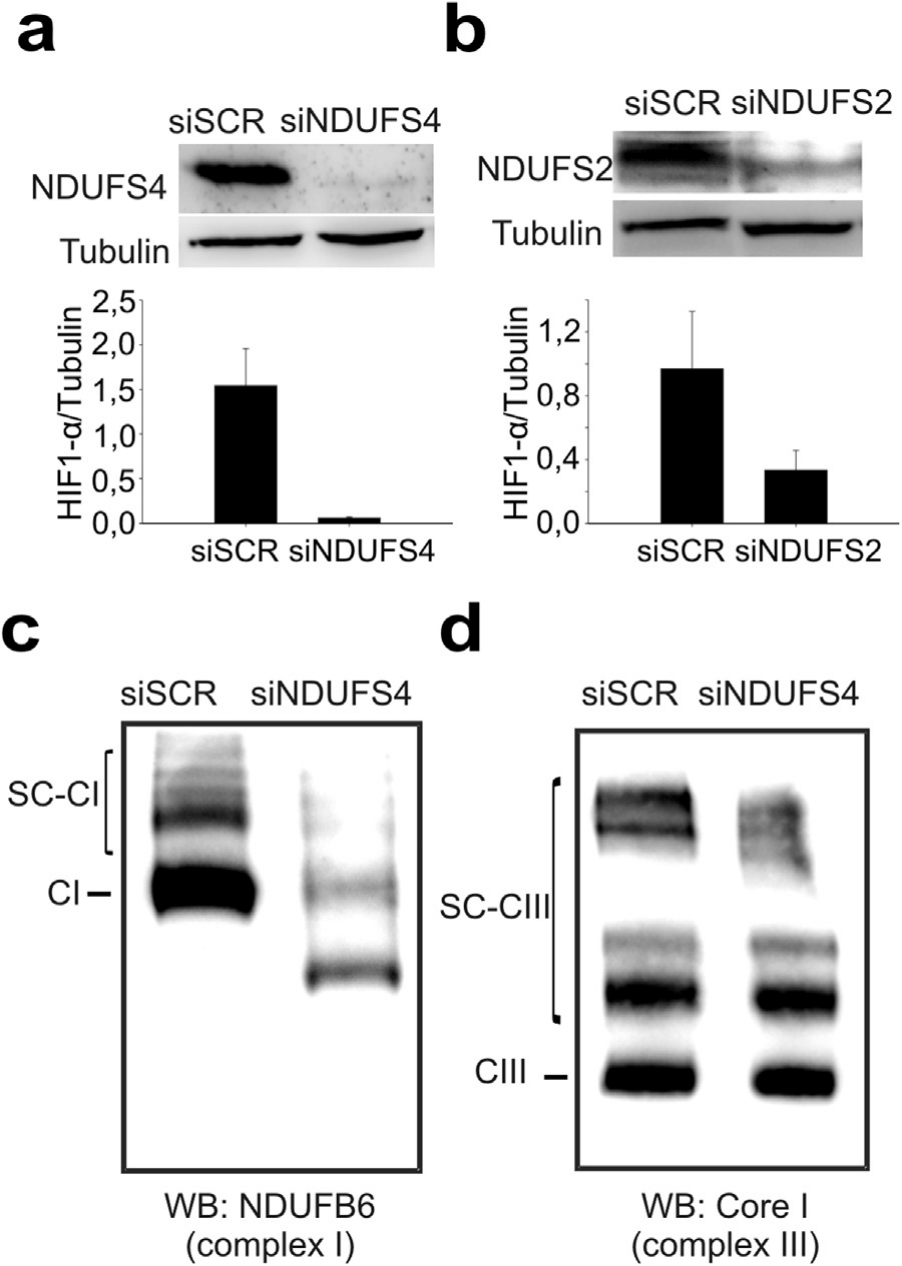
**Silencing of complex I subunits specifically affects the assembly of complex I-containing supercomplexes** (a and b) Protein extracts from BAECs treated with siSCR, siNDUFS4 or siNDUFS2 were immunoblotted for NDUFS4 or NDUFS2 proteins with tubulin as loading control. Up: representative image; down: quantification of three independent experiments (mean±s.e.m.). (c and d) BN-PAGE of siSCR-treated or siNDUFS4-treated BAECs, analyzed by western blotting with antibodies against NDUFB6 (complex I; c) or Core I (complex III; d). Representative image of three independent experiments.

**Fig. 2:**
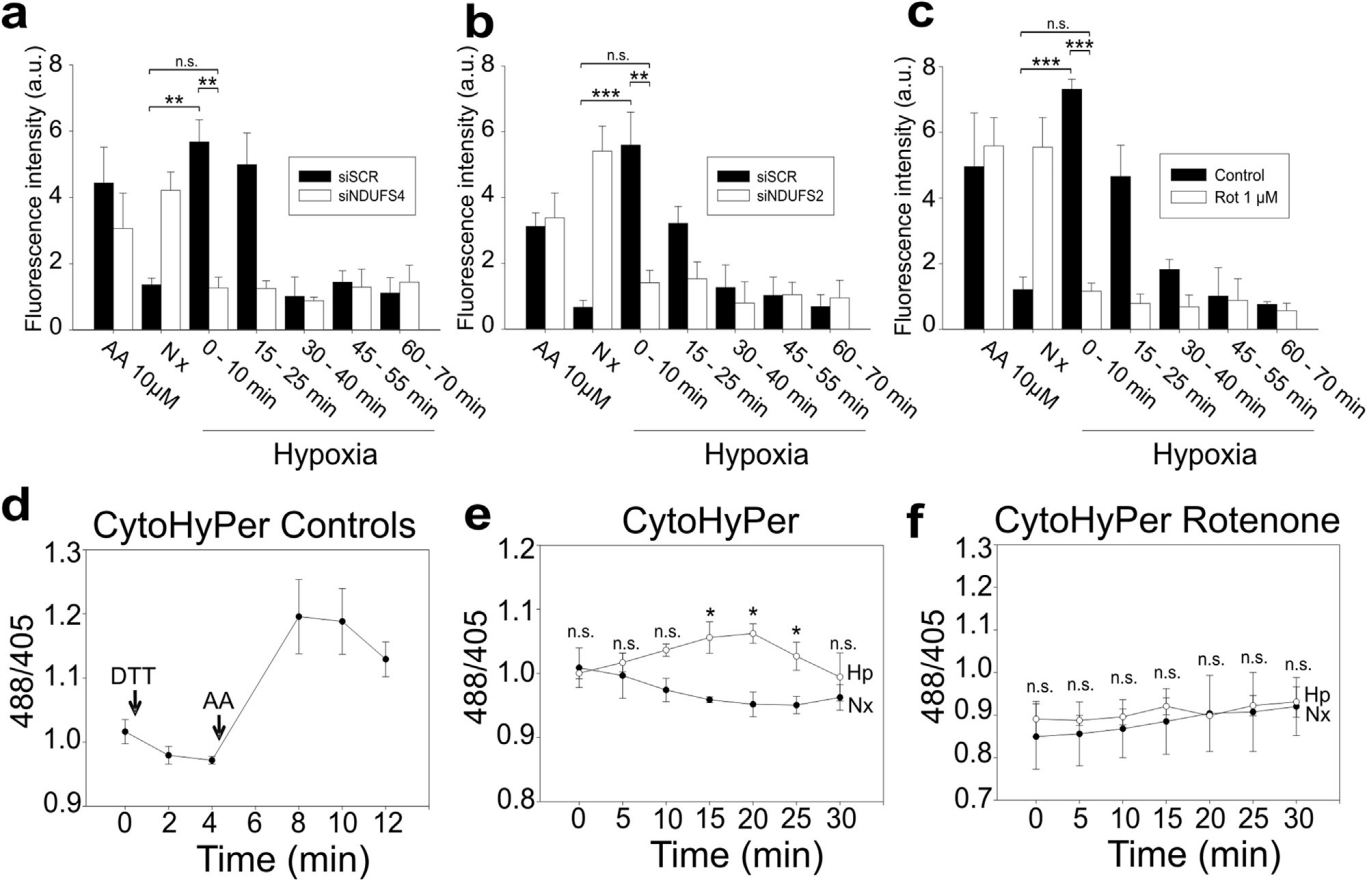
**Interference or inhibition of complex I prevent the increase in ROS production triggered by hypoxia.** (a-c) Detection of superoxide production by fluorescence microscopy in fixed cells. Cells were incubated for 60 min in normoxia (Nx), for 30 min in normoxia with antimycin A (AA 10 µM) or incubated with pre-hypoxic medium in a hypoxia chamber at 1% O_2_ (Hp) for 0, 15, 30, 45 or 60 min. DHE (5 µM) was added for additional 10 min and cells were fixed in the hypoxia chamber. (a and b) BAECs were treated with scramble siRNA (siSCR; black bars) or siRNA against NDUFS4 (a) or NDUFS2 (b). (c) BAECs were untreated (Control) or treated with 1 µM rotenone (Rot 1 µM). Data are presented as the mean±s.e.m. of three independent experiments. n.s. non-significant difference, *p<0.05, **p<0.01 and ***p<0.001 (ANOVA with Tukey post hoc test); only the significances between control normoxia and control hypoxia 0–10 min or treated hypoxia 0–10 min groups are shown. (d-f) Detection of ROS by the ratiometric fluorescent protein HyPer. (d) BAECs were transfected with CytoHyPer, treated with 2 mM of dithiothreitol (DTT) and with 30 µM antimycin A (AA). (e and f) CytoHyPer-transfected BAECs either untreated (e) or treated with 1 µM rotenone (f) were subjected to normoxia (Nx, •) or hypoxia (1% O_2_; Hp, ○). Data are presented as the mean±s.e.m. of four independent experiments. n.s. non-significant difference, *p<0.05 Hp vs. Nx (Mann-Whitney *U* test).

We have recently assessed by several methods that different types of cells produce a superoxide burst in the first minutes of the transition from normoxia to hypoxia [32]. In order to analyse the relationship between the superoxide burst in acute hypoxia and complex I function, we silenced in bovine aortic endothelial cells (BAECs) the expression of genes encoding for either an accessory or a core complex I subunit, NDUFS4 and NDUFS2 respectively. We checked the reduction in the amount of each subunit and the stability of mitochondrial complexes and supercomplexes (Fig. 1). NDUFS4 knockdown destabilised complex I and its corresponding supercomplexes including complex III-containing supercomplexes, while preserving isolated complex III and some bands corresponding to other complex III-related supercomplexes (Fig. 1c-d). Both interventions inhibited the superoxide burst in hypoxia (Fig. 2a-b) and increased superoxide levels in normoxia (Fig. 2a-b). We also used rotenone, an inhibitor of complex I, obtaining similar results (Fig. 2c).

We also assessed ROS levels in acute hypoxia using the ratiometric fluorescent protein HyPer. HyPer is reversibly oxidised in cysteine residues due to hydrogen peroxide production; this oxidation alters its fluorescence signal, allowing the detection of changes in the redox state of cell compartments [43]. We transfected BAECs with a targeted cytosolic version of the protein, CytoHyPer, measuring its signal in living cells; the oxidation signal decreased upon treatment with dithiothreitol and increased after mitochondrial ROS production induced by antimycin A (Fig. 2d). Hypoxia induced CytoHyper oxidation during the first twenty minutes, which decreased thereafter (Fig. 2e). This hypoxic ROS production was abolished upon treatment with 1 µM rotenone (Fig. 2f).

### 3.2 Complex I role in the hypoxic superoxide burst does not rely on reverse electron transport or modification by PINK1

**Fig. 3:**
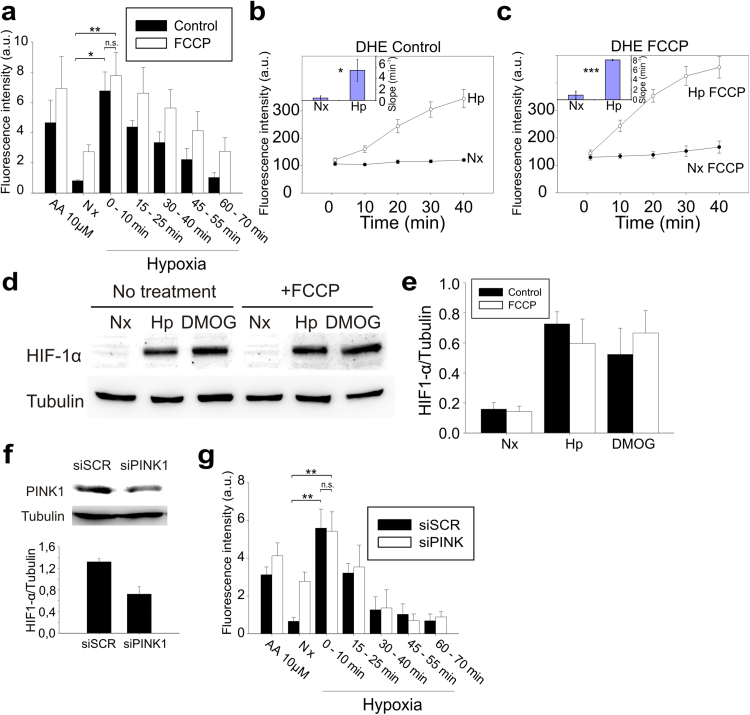
**ROS production in acute hypoxia is not due to reverse electron transport or PINK1 function.** (a) Untreated (black) or BAECs treated with 1 µM FCCP (white) were subjected to the same protocol as in Fig. 2a. Data are presented as the mean±s.e.m. of three independent experiments. n.s. non-significant difference, *p<0.05, **p<0.01 (ANOVA with Tukey post hoc test); only the significances between control normoxia and control hypoxia 0–10 min or treated hypoxia 0–10 min groups are shown. (b and c) Detection of ROS production by live fluorescence microscopy with DHE. BAECs untreated (b) or treated with 1 µM FCCP (c) were subjected to normoxia (Nx, •) or hypoxia (2% O_2_; Hp, ○). (Insets) Slopes considering all time points of each replicate (n=3). The slope for each replicate was estimated by linear regression of the data for all the ROI and time points. Data are presented as the mean±s.e.m. of three independent experiments. n.s. non-significant difference, *p<0.05, ***p<0.001 (Student's *t*-test). (d and e) HIF-1α stabilization measured by western blotting in BAECs treated or not with 1 µM FCCP and exposed for 4 h to normoxia (Nx), normoxia with 1 mM DMOG or to hypoxia (1% O_2_, Hp). Tubulin was used as loading control. (d) Representative images; (e) quantification of three independent experiments (mean±s.e.m.). (f) Protein extracts from BAECs treated with siSCR or siPINK1 were immunoblotted for PINK1 protein with tubulin as loading control. Up: representative image; down: quantification of three independent experiments (mean±s.e.m.). (g) BAECs were treated with scramble siRNA (siSCR; black bars) or siRNA against PINK1 (white bars) and subjected to the same protocol as in Fig. 2a. Data are presented as the mean±s.e.m. of three independent experiments. n.s. non-significant difference, **p<0.01 (ANOVA with Tukey post hoc test); only the significances between control normoxia and control hypoxia 0–10 min or treated hypoxia 0–10 min groups are shown.

We next wanted to assess the mechanism by which complex I is involved in the ROS burst in acute hypoxia. Reverse electron transport (RET) in complex I (from ubiquinol to NAD^+^) is associated with high ROS production after ischemic accumulation of succinate [27], the substrate of cII. Thus, we explored whether RET was required for the superoxide burst in the transition from normoxia to hypoxia through two different approaches. Since superoxide production in complex I reverse mode, either by complex I itself or by dehydrogenases from the TCA cycle, relies on high mitochondrial membrane potential, which can be easily abolished by treatment with an OXPHOS uncoupler [24], [44], we treated BAECs with the uncoupler FCCP, finding that the superoxide burst was barely affected (Fig. 3a). We also analyzed superoxide production in the transition from normoxia to hypoxia by live imaging with DHE [32], finding that FCCP did not reduce the increase in the probe signal (Fig. 3b-c).

Since the production of mitochondrial ROS in hypoxia is associated with hypoxia-inducible factor 1α (HIF-1α) stabilization [30], [32], we reasoned that if RET was operating under hypoxia FCCP should abolish it. However, treatment with FCCP had no effect on HIF-1α stabilization in normoxia or in hypoxia (Fig. 3d-e). These results show that reverse electron transport through complex I is not the main mechanism of superoxide production in acute hypoxia.

PINK1 mutation has been associated with alteration in complex I function [22] and with ROS production and HIF-1α stabilization in hypoxia [23]. Therefore, we wondered whether PINK1 was involved in the hypoxic superoxide burst. We silenced the expression of PINK1 in BAECs (Fig. 3f) and observed that the ROS levels in normoxia were higher than in the non-interfered cells, but they still rose in the transition to hypoxia (Fig. 3g). Thus, PINK1 is not involved in the superoxide burst in acute hypoxia.

### 3.3 Acute hypoxia deactivates mitochondrial complex I

Complex I exists in two conformations that are associated with catalytically active (A) or inactive -‘deactive’- (D) forms [17], [20]. A/D transition can be induced *in vitro* by thermal deactivation since complex I deactivates with the lack of substrates. In addition, a physiological stimulus shown to trigger A/D transition is prolonged ischemia or anoxia [20], [45], therefore we wondered whether acute hypoxia could also promote deactivation of complex I.

**Fig. 4:**
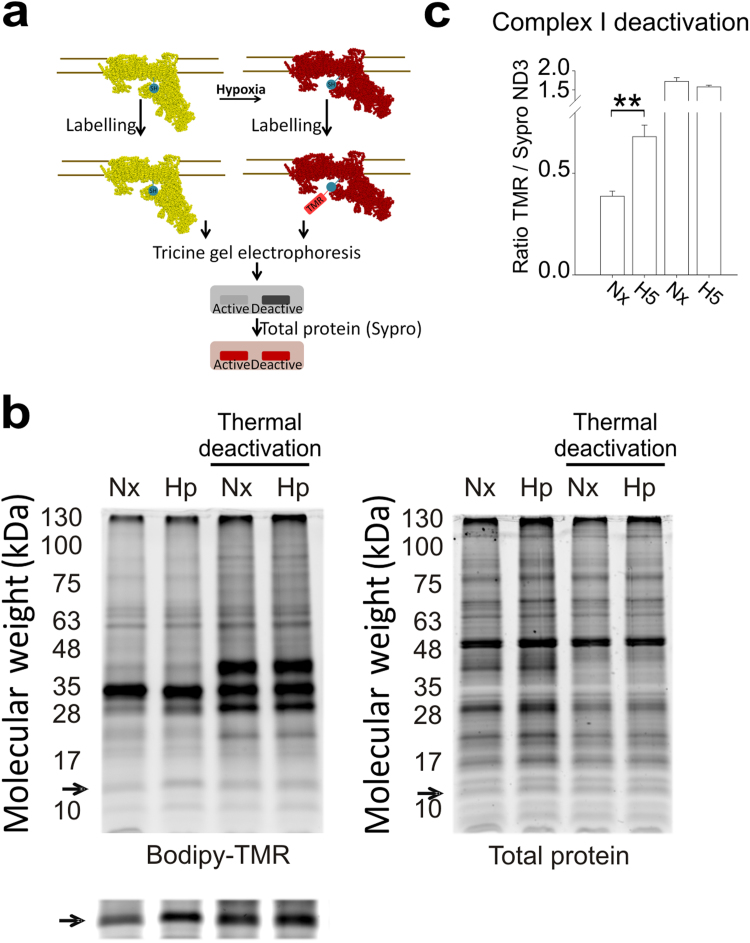
**Acute hypoxia deactivates complex I in BAECs.** (a) Cys-39 of ND3 remains buried in active complex I (yellow), while it is exposed in deactive complex I (red). Mal-Bodipy-TMR was used to label exposed Cys before electrophoretic protein separation. TMR fluorescence signal for the ND3 band was higher when complex I was deactive (grey picture). Protein amount for the same band is detected with Sypro Ruby staining (red picture). (b, c) Mitochondrial membranes from BAECs treated for 5 min in normoxia (Nx) or hypoxia (1% O_2_, Hp or H5) were split in two equal parts; one part was incubated for 1 h at 37 °C to fully deactivate complex I (Thermal deactivation), whereas the other was kept on ice. (b) Bodipy-TMR signal reflects exposed Cys (left) and Sypro Ruby signal detects total protein (right). Arrows (➙) mark the band corresponding to ND3 identified by LC-MS/MS; the lower image on the left is a more exposed photograph of the Bodipy-TMR signal. (c) Band corresponding to TMR-labelled ND3 was quantified and normalized to total ND3. Data are presented as the mean±s.e.m. of six independent experiments. n.s. non-significant difference, **p<0.01 H5 vs. Nx (Mann-Whitney *U* test). (For interpretation of the references to color in this figure legend, the reader is referred to the web version of this article).

**Fig. 5:**
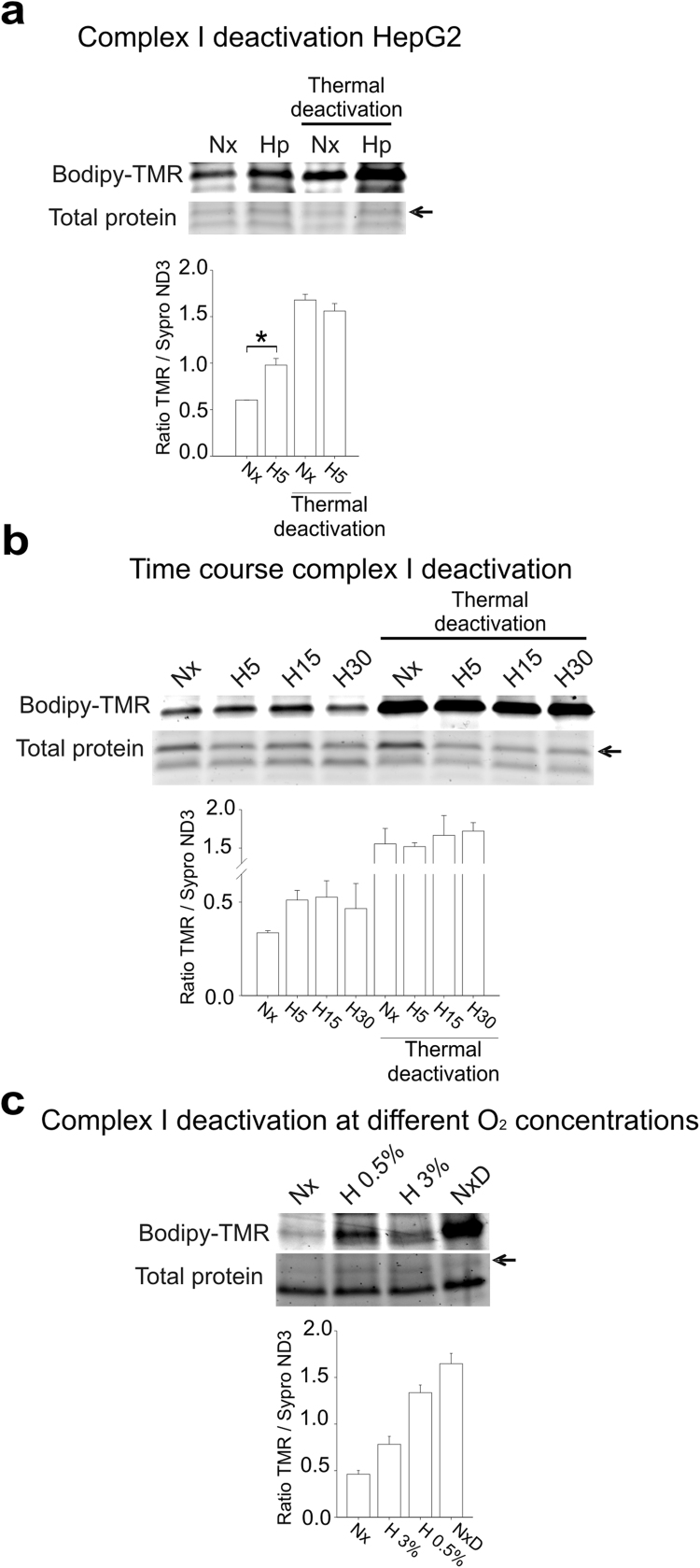
**Acute hypoxia deactivates complex I**. (a) Mitochondrial membranes from HepG2 treated as in Fig. 4. Up: representative image; down: quantification of ND3 Cys exposure (mean±s.e.m. of four independent experiments). *p<0.05, H5 vs. Nx (Mann-Whitney *U* test). (b) Mitochondrial membranes of BAECs treated for 5 min in normoxia (Nx) or in hypoxia (1% O_2_) for 5 (H5), 15 (H15) or 30 min (H30) and treated as in Fig. 4. Up: representative image showing; down: quantification of ND3 Cys exposure (mean±s.e.m. of three independent experiments). (c) Mitochondrial membranes of BAECs subjected to normoxia or different hypoxia conditions (3% or 0.5% O_2_) for 5 min; NxD: thermal deactivation of normoxic sample. Up: representative image; down: quantification of ND3 Cys exposure (mean±s.e.m. of four independent experiments). Arrows (➙) mark the band corresponding to ND3 in total protein.

**Fig. S1:**
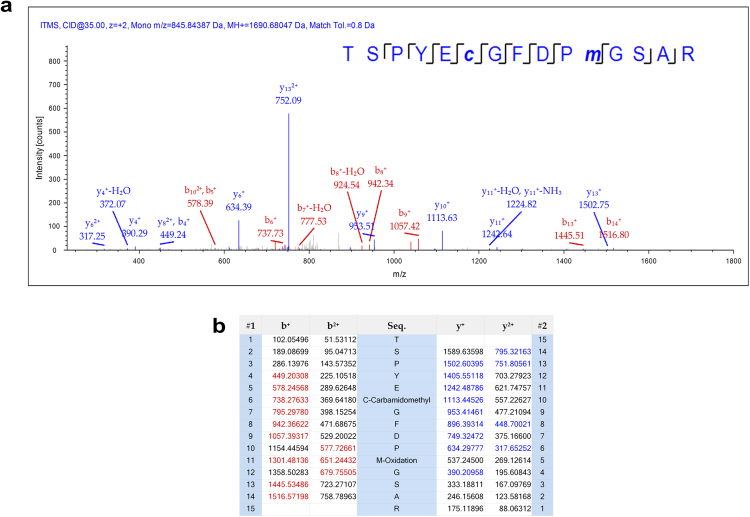
**Identification of ND3 band.** (a and b). MS/MS identification of a peptide of bovine ND3 containing Cys-39, obtained from tryptic digestion of the ND3 band of a gel from the experiment in Fig. 5b. CID fragmentation MS/MS spectrum (a) and table (b) indicating the assigned fragments from the b and y series are shown.

A/D transition involves a conformational rearrangement of complex I resulting in the exposure of Cys-39 within the bovine mitochondrial subunit ND3 [16], [46]. Labelling of this thiol group with thiol-specific reagents thus constitutes a suitable marker for deactivation of complex I [18], [19], [46]. We attempted to detect the exposed Cys-39 residue of ND3 using a fluorescent maleimide reagent under non-denaturing conditions. Using this technique, exposed cysteines were labelled in mitochondrial membrane preparations from bovine aortic endothelial cells (BAECs) subjected to normoxia or hypoxia, allowing the detection of both the exposed Cys labelling and total protein signal for each electrophoretic band in the same gel (Fig. 4a-b). The exposed Cys signal of a protein species of approximately 10 kDa was clearly increased after thermal deactivation [19], [46] and, more interestingly, in samples from hypoxia-treated BAECs (Fig. 4b, left and bottom). The total protein signal of that band was similar in all gel lanes (Fig. 4b, right). Mass spectrometry analysis of this band identified a peptide of ND3, confirming that this band corresponded to ND3 (Supplementary Fig. 1). Quantification of the ratio of TMR/Sypro signals (exposed Cys vs total protein) for this band clearly showed that Cys-39 (the only Cys of this protein) was more exposed after 5 min of hypoxia in BAECs (Fig. 4c) or in the hepatocyte cell line HepG2 (Fig. 5a). Furthermore, increased exposure of ND3 Cys-39 was maintained from 5 to 30 min of hypoxia (Fig. 5b) and increased gradually with decreasing oxygen tension (Fig. 5c).

**Fig. 6:**
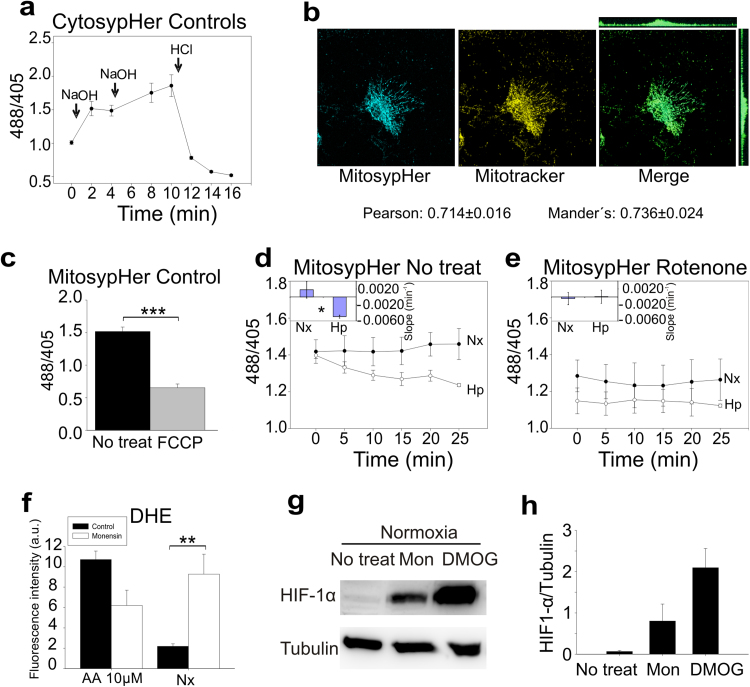
**Acute hypoxia enhances complex I Na**^**+**^**/H**^**+**^**antiporter activity.** (a) A cytosolic version of sypHer was transfected in BAECs to analyse pH change in control conditions. BAECs were treated with two subsequent additions of 30 µM NaOH and one of 8 mM HCl. Data are represented as mean±s.e.m. of eight different ROIs. (b) BAECs transfected with mitosypHer were incubated with 25 nM MitoTracker CMTMRos for 20 min and fixed. Representative fluorescence confocal microscopy images show mitochondrial localisation of mitosypHer. Estimated Pearson and Mander's correlation coefficients for colocalisation of both signals are shown. (c) 488/405 signals ratio reflecting intramitochondrial pH in BAECs transfected with mitosypHer either untreated (No treat) or treated with 1 µM FCCP (FCCP). Data are represented as mean±s.e.m. of five independent experiments. ***p<0.001 (Student's *t*-test). (d and e) Intramitochondrial pH measured with mitosypHer by live confocal microscopy in BAECs either untreated (d) or treated with 1 µM rotenone (e) and subjected to normoxia (Nx, •) or hypoxia (1% O_2_; Hp, ○). Data are represented as mean±s.e.m. of the ratio between the fluorescence signals with excitation at 488 nm and 405 nm of four independent experiments. (Insets) Slopes considering all time points of each replicate (n=4) are plotted as mean±s.e.m. The slope for each replicate was estimated by linear regression of the data for all the ROI and time points. *p<0.05 (Student's *t*-test). (f) Non treated BAECs or treated with 10 µM monensin for 30 min in normoxia were subjected to the same procedure as in Fig. 2a. Data are represented as mean±s.e.m. of three independent experiments. **p<0.01 (ANOVA with Tukey post hoc test); only the significance between non-treated normoxia and monensin-treated normoxia is shown. (g) HIF-1α stabilization measured by western blotting in BAECs treated or not with 10 µM monensin (Mon) or with 1 mM DMOG and exposed for 4 h to normoxia (Nx). Tubulin was used as loading control. Representative images of three independent experiments are shown. (h) Quantification of (g); mean±s.e.m. of three independent experiments.

Since the deactive form of complex I is associated to a Na^+^/H^+^ antiporter (NHE) activity we wondered whether this could be the situation under acute hypoxia. Originally, the antiporter activity of the deactive form of complex I was measured by changes in *Bos taurus* heart submitochondrial particles (SMPs) ΔpH [18]. We measured mitochondrial matrix pH as a readout of NHE activity in BAECs transfected with the ratiometric mitochondrial pH indicator mitosypHer [47]. We validated the ability of the fluorescent protein to respond to pH changes (Fig. 6a), the subcellular localisation of its mitochondria-targeted version (Fig. 6b) and its ability to measure mitochondrial matrix acidification after incubation of cells with FCCP, an uncoupler of the OXPHOS system (Fig. 6c). Acute hypoxia acidified the mitochondrial matrix (Fig. 6d). Acidification was abolished by rotenone, a Q-site complex I inhibitor (Fig. 6e). Although this approach does not exclude other mechanisms that could also contribute to matrix acidification, the results are compatible with the hypothesis of an increase in the NHE activity of complex I.

Taken together, these results show that acute hypoxia induces complex I deactivation, exposing ND3 Cys-39, and acidifies the mitochondrial matrix probably by enhancing its NHE activity.

In order to assess whether Na^+^/H^+^ exchange could trigger ROS production and imitate the hypoxic response, we treated BAECs with the Na^+^/H^+^ exchanger monensin in normoxia. This treatment clearly increased ROS production (Fig. 6f) and also stabilised HIF-1α in normoxia (Fig. 6g-h), strongly suggesting that Na^+^/H^+^ exchange by deactive complex I could be a mechanism involved in ROS signalling in acute hypoxia.

### 3.4 Complex I deactivation is involved in the hypoxic response in neuronal cells and brain tissue

**Fig. 7:**
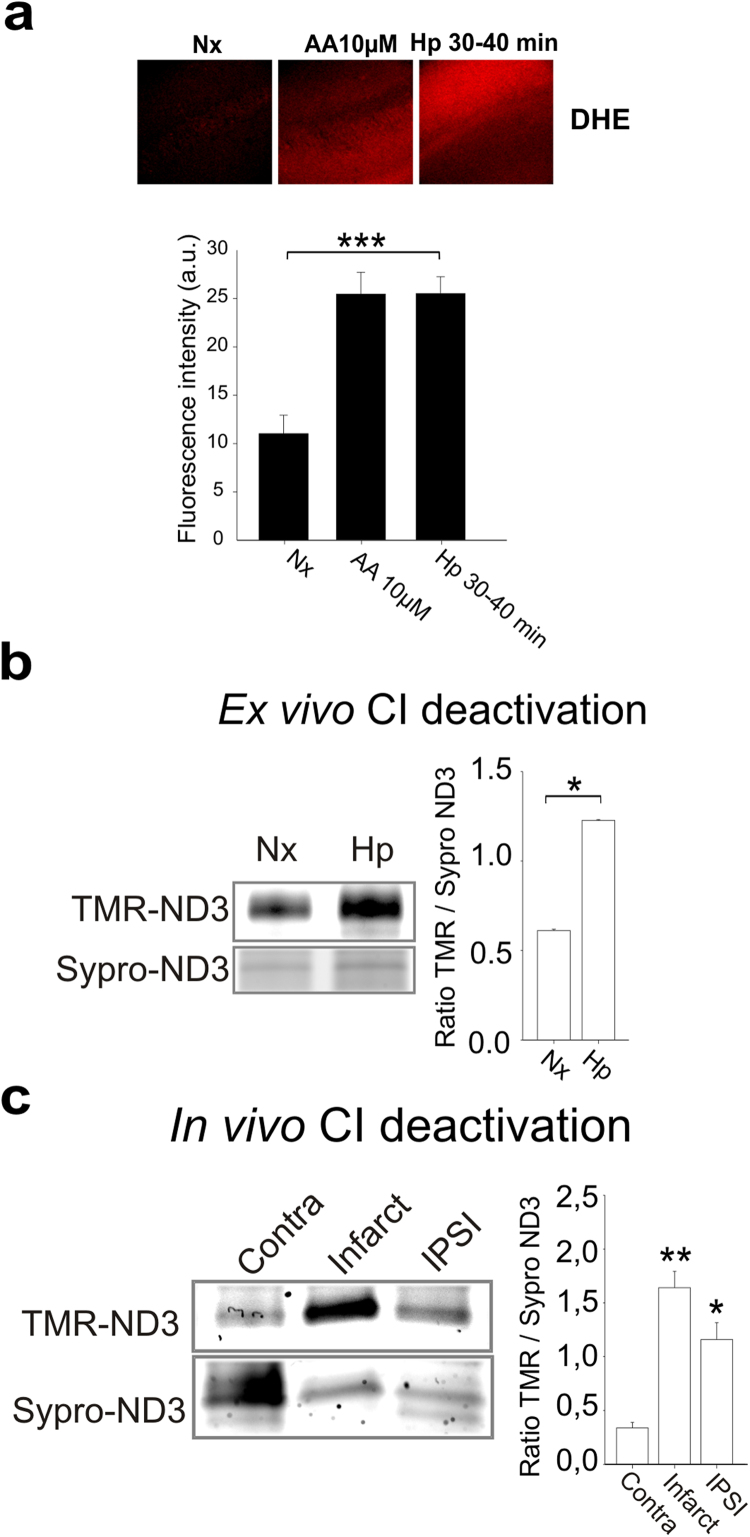
**Complex I deactivation correlates with ROS production and occurs*****ex vivo*****and*****in vivo***. (a) Hippocampal slices were incubated for 30 min in normoxia (Nx), in normoxia with antimycin A (AA 10 µM) or in hypoxia at 1% O_2_ (Hp 30–40 min). DHE (5 µM) was added for additional 10 min, and slices were fixed in the hypoxia chamber. (upper panel) Representative images show DHE fluorescence. (lower panel) Quantification of DHE fluorescence signal. Data are presented as mean±s.e.m. of four independent experiments. ***p<0.001 (ANOVA with Tukey post hoc test); only the significance between Nx and Hp 30–40 min is shown is shown. (b) Hippocampal slices were subjected to 30 min of normoxia (Nx) or hypoxia (1% O_2_; H30) and treated as in Fig. 4. Left: representative image; right: Quantification of ND3 Cys exposure (mean±s.e.m. of three independent experiments). *p<0.05 (Mann-Whitney *U* test). (c) Mice were subjected to photothrombotic stroke induction and ND3 Cys exposure was estimated as in Fig. 4 from samples of different regions of the brain: infarct, ipsilateral (IPSI) and contralateral (Contra). Left: representative image; down: quantification of ND3 Cys exposure (mean±s.e.m. of three independent experiments). *p<0.05, **p<0.01 vs. Contra (ANOVA with Tukey post hoc test).

Since most of the preceding experiments were carried out in non-excitable primary endothelial cells, we questioned whether the hypoxic superoxide burst and complex I deactivation could also be observed in excitable cells, such as neurons. For this, we used an *ex vivo* model: we exposed isolated mouse hippocampal slices to acute hypoxia, finding increased superoxide levels after 30 min (Fig. 7a). In this condition, complex I is deactivated (Fig. 7b), suggesting its implication in ROS production.

Finally, we addressed whether complex I deactivation could occur in an *in vivo* model of photothrombotic ischemic injury. Complex I deactivation was clearly observed in the infarcted area of mice subjected to an ischemic insult and, interestingly, also in the ipsilateral area, the area where reduced blood flow arrives (Fig. 7c). Overall, these results highlight the relationship of complex I deactivation and ROS production, in neurons in *ex vivo* and *in vivo* models.

## 4 Discussion

There has been a long-standing debate about the increase or decrease in ROS levels in response to hypoxia. We have recently shown by different methodologies that superoxide levels are increased for a limited time in the response of different cell types to acute hypoxia, what we called a superoxide burst [32]. Recently, it has been shown that mitochondrial complex I is involved in acute oxygen sensing by the glomus cells of the carotid body through ROS production [34]. Since carotid body is a very specialised tissue and could have a unique machinery for oxygen sensing, we wondered whether complex I was also necessary for ROS production in other cell types and tissues. By means of pharmacological and genetic inhibition, we show that complex I is involved in ROS production in endothelial cells under acute hypoxia. Interestingly, with these interventions the ROS levels correlate with the oxygen concentration, with a high ROS production in normoxia that diminishes in the transition to hypoxia.

We have further analyzed different properties of complex I that could be implicated in the mechanism triggering the hypoxic superoxide burst. Complex I can produce ROS by both forward and reverse reactions, and the latter (reverse electron transfer, RET) is implicated in ROS production in reperfusion [27]. RET relies on large amounts of succinate which reduce the pool of ubiquinone and drive electrons through complex I when the mitochondrial inner membrane is hyperpolarised [24], [25]. Treatment with OXPHOS uncouplers, such as FCCP, depolarise mitochondria so that the electron transport chain can operate at maximal capacity and RET is abolished [24], [44]. However, the superoxide burst in endothelial cells was not abolished after treatment with FCCP, neither in fixed cells or live imaging experiments (Fig. 3a-c). This suggests that RET is not the mechanism triggering superoxide production in acute hypoxia.

We also hypothesised that PINK1 could have a role in altering complex I in the transition from normoxia to hypoxia. It has been recently described that PINK1 regulates ecomplex I [22] and its deficiency in neurons produces higher ROS and HIF-1α stabilization in normoxia [23]. When we interfered PINK1 expression, we also observed higher ROS levels in normoxia, but a clear increase remained in the first minutes of hypoxia (Fig. 3g), discarding PINK1 as a key component in triggering the superoxide burst.

It should be noted that ROS detection has some limitations that need to be considered. Although we have previously specifically detected that superoxide anion is produced in the first minutes of hypoxia [32] we cannot discard that the interventions made in this paper alter the specific reaction of DHE and superoxide, by altering for example redox intermediates. Therefore, in this case DHE could behave as a general ROS indicator rather than a specific superoxide probe [42]. In addition, HyPer fluorescent proteins detect changes in ROS levels by oxidoreduction of Cys 199 thiol group which promotes a shift in their spectral properties [43]. Since oxidation of thiols can be achieved by several oxidants, including hydrogen peroxide or peroxynitrite, we cannot identify which ROS is specifically being detected by HyPer. Thus, although we cannot point out which ROS is detected in each case, the increase in ROS production in acute hypoxia and its modulation by complex I presence and function is clear from the experiments presented herein.

Another intrinsic property of complex I that we have explored in relation with ROS production is A/D transition. We have been able to detect complex I deactivation by labelling the thiols in the submitochondrial particle (SMPs) samples with a novel protocol adapted from Galkin et al. [19]. Upon deactivation a conformational change rearranges several subunits of complex I and Cys39 of ND3 becomes exposed [16], [19]. We label exposed thiols in the SMPs samples with a fluorescent maleimide, so that the difference in fluorescence intensity correlates with increased exposure of ND3-Cys39 reflecting complex I deactivation (Fig. 4a). Thus, we observed a clear increase in ND3 exposure upon thermal deactivation, and a partial increase starting at 5 min of hypoxia (1% O_2_), probably as the start of deactivation, in consistence with previous reports that showed that a more prolonged ischemia or anoxia (at least 20 min) was necessary to deactivate complex I. Interestingly, the degree of complex I deactivation negatively correlated with the amount of oxygen, showing that progressive oxygen depletion is sufficient to induce complex I deactivation, starting from a rather slight hypoxia (3% O_2_). Of note, in the stroke model, we found a profound deactivation of complex I in the anoxic infarcted area, while the hypoxic ipsilateral area showed partial complex I deactivation, correlating with results obtained in cell culture. Interestingly, it has been recently shown that greater complex I deactivation in brain cells correlated with higher ROS levels [48].

Complex I deactivation could trigger ROS production by several means. It would imply an arrest in NADH consumption and its accumulation; this could reverse the reaction of the tricarboxylic acid (TCA) cycle dehydrogenases which are known to produce ROS by this reaction [49]. On the other hand, complex I A/D transition could modify ubiquinone pool redox state and trigger the production of superoxide by complex III; this would be in accordance with previous reports showing the involvement of complex III in hypoxic ROS production [30]. Another possible explanation arises from complex I switch to a Na^+^/H^+^ antiporter.

We propose that this activity could be involved in superoxide production by mitochondria; this hypothesis is supported by the fact that treatment with the Na^+^/H^+^ antiporter monensin increased superoxide levels and HIF-1α stabilization (Fig. 6f and g). From the energetic point of view, Na^+^/H^+^ antiporter activity would dissipate the H^+^ gradient across the mitochondrial inner membrane without affecting ΔΨmt. Given that ΔΨmt represents around 90% of the Δµmt [50], [51] and that among the OXPHOS complexes only deactive complex I is permeable to Na^+^, the Na^+^/H^+^ antiporter activity would serve to maintain ΔΨmt removing ΔpH. This could, for instance, keep complex V unable to depolarise mitochondria by translocating H^+^ back to the matrix. Alteration of the Na^+^ gradient could directly affect other ROS-producing complexes or influence mitochondrial ion homeostasis which could have consequences on superoxide production [48], although this requires further investigation.

This hypothesis is still mainly correlative and we have not provided a definitive causal proof. Of note, we are not aware of any procedure or complex I mutant form capable to exacerbate or inhibit deactivation or Na^+^/H^+^ antiporter activity, without affecting the rest of activities of complex I. The mechanism underlying A/D transition in hypoxia is not well known and whether it is intrinsic to complex I or involves other cellular components remains to be discovered. In addition, since it has been proposed that the pumping H^+^ subunits in complex I are the ones that carry out Na^+^/H^+^ exchange [18] and these subunits are encoded by the mitochondrial DNA, it is not possible to modify the expression of such subunits specifically. Therefore, we can only suggest the link between deactivation and ROS production, since it is not possible to provide an actual connection through mutagenesis studies or genetic manipulation.

In addition, it is not known whether hypoxia is the primary stimulus deactivating complex I (in that case, it would behave as an oxygen sensor), or if it needs the participation of other players. Proteins, lipids and ions [21], [22], [52] regulate complex I activity, thus different tissues, physiological conditions and diseases can influence complex I activation status, which is predicted to have important consequences from the point of view of bioenergetics and ROS production. Given that complex I deficiency underlies several diseases, and hypoxia and anoxia are present in many physiological and pathophysiological scenarios, the study of the influence of complex I deactivation in mitochondrial ROS production in hypoxia becomes more important as a new player in mitochondrial homeostasis.

## Conflicts of interest

The authors declare no conflict of interest.
